# Long COVID: Clinical characteristics, proposed pathogenesis and potential therapeutic targets

**DOI:** 10.3389/fmolb.2023.1157651

**Published:** 2023-04-26

**Authors:** Grace Kenny, Liam Townsend, Stefano Savinelli, Patrick W. G. Mallon

**Affiliations:** ^1^ Centre for Experimental Pathogen Host Research, University College Dublin, Dublin, Ireland; ^2^ Department of Infectious Diseases, St Vincent’s University Hospital, Dublin, Ireland

**Keywords:** SARS-CoV-2, long COVID, post-acute sequelae of SARS-CoV-2 infection, PASC, post COVID condition

## Abstract

The emergence of persistent ill-health in the aftermath of SARS-CoV-2 infection has presented significant challenges to patients, healthcare workers and researchers. Termed long COVID, or post-acute sequelae of COVID-19 (PASC), the symptoms of this condition are highly variable and span multiple body systems. The underlying pathophysiology remains poorly understood, with no therapeutic agents proven to be effective. This narrative review describes predominant clinical features and phenotypes of long COVID alongside the data supporting potential pathogenesis of these phenotypes including ongoing immune dysregulation, viral persistence, endotheliopathy, gastrointestinal microbiome disturbance, autoimmunity, and dysautonomia. Finally, we describe current potential therapies under investigation, as well as future potential therapeutic options based on the proposed pathogenesis research.

## Introduction

### Identification of long COVID as clinical syndrome

The clinical spectrum of COVID-19, the disease caused by severe acute respiratory syndrome coronavirus 2 (SARS-CoV-2) infection, has been extensively characterised (Huang et al., 2020a; Xu et al., 2020). The majority of affected individuals experience a mild or moderate disease phenotype, with fewer than 5% developing critical illness. In the aftermath of the first wave of infection, there were increasing reports of persistent ill-health beyond resolution of acute infection. These ongoing symptoms were protean, with fatigue, cognitive dysfunction, and respiratory symptoms most commonly reported (Carfì et al., 2020; Townsend et al., 2020). Up to one-fifth of those who have ever had COVID report symptoms of long COVID (USA DoHaHS, 2022). Many experience disabling symptoms, with 44% of those with long COVID in one survey unable to return to work (Cutler, 2022) Post-viral conditions following other infections have been previously described. Indeed, the assessment of the long-term consequences of the severe acute respiratory syndrome (SARS) epidemic (caused by the novel coronavirus SARS-CoV) found that a subset (51%) of those affected in Toronto experienced persistent fatigue, diffuse myalgias, weakness and depression 1 year after their acute illness and could not return to work (Moldofsky and Patcai, 2011). In a similar follow-up study amongst 233 SARS survivors in Hong Kong, over 40% of respondents reported ongoing fatigue 40 months after infection (Lam et al., 2009). In those affected by the subsequent Middle-Eastern Respiratory Syndrome Coronavirus (MERS-CoV) outbreak in Korea, prolonged symptoms and fatigue were reported up to 18 months after acute infection (Lee et al., 2019). However, no pathological mechanism underlying these symptoms has been described.

Post-infectious fatigue syndromes have been reported following a myriad of infections other than coronaviruses, including Epstein-Barr Virus (EBV), Q-Fever and Ross River Virus (RRV) infections, as well as rickettsiosis (Hickie et al., 2006). Similar to post-coronavirus illnesses, no definite mechanism has been identified for the development of these syndromes. An area of prior focus in these conditions has been the autonomic system, with post-infectious dysautonomia proposed as an underlying mechanism to explain commonly reported post-infectious symptoms such as abnormal heart rate and blood pressure responses to physiological stressors, most notably postural orthostatic tachycardia syndrome (POTS). Interestingly, prior studies in the area of chronic fatigue syndrome/myalgic encephalomyelitis (CFS/ME) have also shown a variety of changes in autonomic function, but a clear unifying pathological basis to explain the emergence of dysautonomia post infection is lacking (Newton et al., 2007).

A common stumbling block for mechanistic studies into post-infectious fatigue and reduced exercise tolerance is an absence of clear timing of onset from the purported triggering infection. The COVID-19 pandemic has provided an opportunity to study physical sequalae and their underlying mechanisms following acute viral infection in a setting seldom found: a large number of individuals with confirmed infection by a known virus at a known timepoint. Another challenge lies in how to appropriately characterise long COVID, given its heterogenous nature and the wide number of symptoms reported. There are various terms and clinical definitions from different guidance bodies used to classify the constellation of symptoms and incident conditions that occur following acute COVID-19, including ‘post COVID-19 syndrome’, ‘post COVID-19 condition’ and ‘post-acute sequelae of COVID-19’ (PASC) (Venkatesan, 2021; WHO, 2021; Centers for Disease Control and Prevention, 2022). Long COVID is the term coined by those who are experiencing this condition, and more recently the CDC have incorporated this label as the working definition in its National Research Action Plan. This working definition defines long COVID as signs, symptoms or conditions that develop after SARS-CoV-2 infection and are present four or weeks after the initial infection (USA DoHaHS, 2022). However there is heterogeneity in definitions used in studies, with some requiring symptoms to persist for eight or 12 weeks (Sudre et al., 2021; Subramanian et al., 2022). In this review we outline the range of symptoms encompassed by this condition, the leading hypotheses of the pathophysiologic basis for this condition, research gaps and future directions.

## Clinical spectrum of long COVID

### Long COVID phenotypes

**FIGURE 1:**
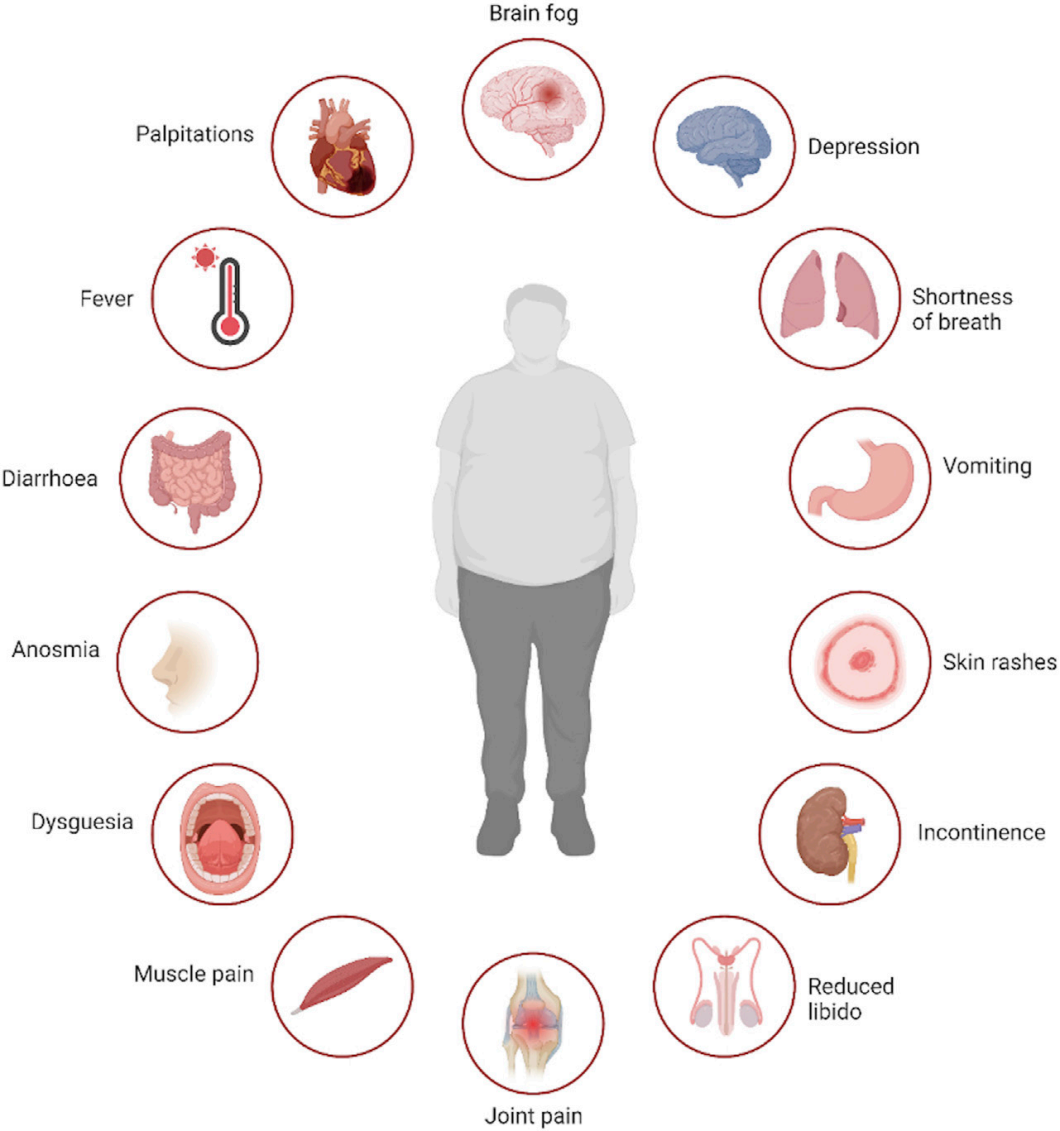
Multisystem symptoms associated with *long COVID*. Legend: Selection of symptoms reported by patients suffering with long COVID, demonstrating the multi-system nature of the disease. Created with BioRender.com.

Symptoms of long COVID span multiple physiological domains, with more than 100 different symptoms reported (Subramanian et al., 2022), examples of which are shown in Figure 1. Given the heterogeneity in post COVID sequelae and the breadth of symptoms reported, a major challenge in studying long COVID is characterising distinct phenotypes that can then assist with the selection of appropriate cohorts of individuals and controls to include in translational studies or therapeutic trials of specific agents. This is particularly challenging in the large cohort of individuals who, despite debilitating symptoms, have normal routine clinical investigations (Sneller et al., 2022). Further complicating the matter, older individuals who were hospitalised during the acute phase of the COVID-19 illness are overrepresented in many studies to date (Littlefield et al., 2022; Su et al., 2022), making it difficult to untangle abnormalities expected to occur after a severe systemic illness, which might disproportionately affect older adults, from those directly related to SARS-CoV-2. Additionally, with the exception of smell and taste disorders, the most commonly reported long COVID symptoms are all relatively frequent in the general population (Subramanian et al., 2022), and a relapsing and remitting pattern of symptoms is common (Davis et al., 2021). A number of studies have attempted to overcome the heterogeneity of this condition by correlating individual immune abnormalities with specific symptoms, but such multiple comparisons increases the risk of chance findings and potentially erroneous conclusions.

**FIGURE 2:**
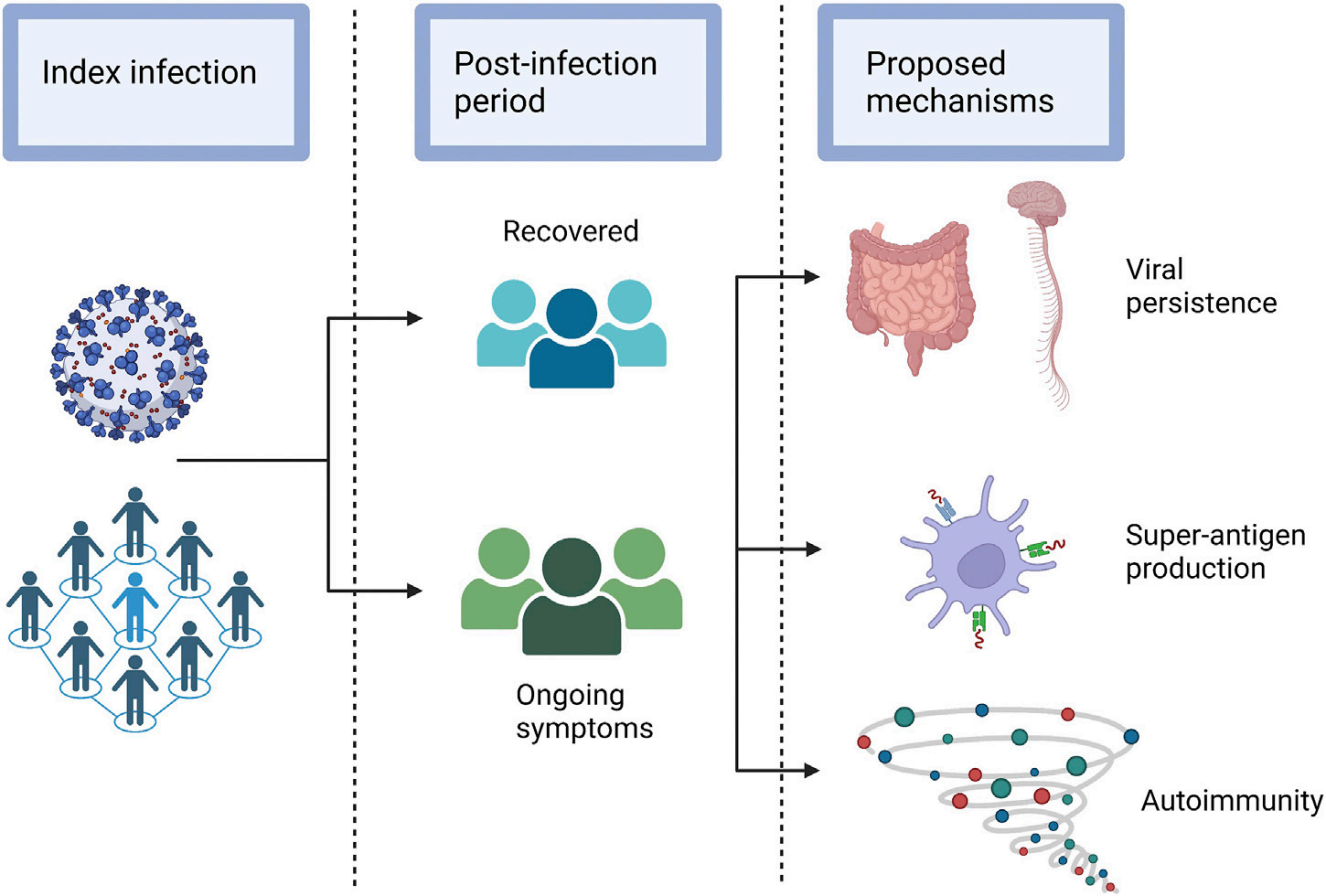
Hypothesised sequelae of SARS-CoV-2 that lead to long COVID. Legend: A selection of the possible sequelae of SARS-CoV-2 that may lead to the various post-acute pathologies that occur in a subset of individuals.

Identifying distinct long COVID clinical phenotypes or syndromes that may have unique underlying pathophysiologic mechanisms (Figure 2) has been the focus of a number of studies. Some have proposed a manual subgrouping of symptom profiles or grading of symptom severity, while others have used unsupervised statistical methods to identify data driven phenotypes appropriate for this novel condition. While these studies differ in terms of cohorts, data collection methods, symptoms included and analytic approaches, four common themes across studies are emerging.

The phenotype most commonly reported across studies using unsupervised clustering is a cardiorespiratory phenotype (Kenny et al., 2022a; Zhang et al., 2022a; Canas et al., 2022; Caspersen et al., 2022; Danesh et al., 2022; Frontera et al., 2022; Sahanic et al., 2022; Whitaker et al., 2022; Reese et al., 2023), with breathlessness reported as a predominant symptom, while cough, chest pain and palpitations are reported to a varying degree. Similarly, a number of studies have identified a cluster with a high number of symptoms, including pain and musculoskeletal symptoms such as joint pain and myalgia (Kenny et al., 2022a; Zhang et al., 2022a; Canas et al., 2022; Fernández-de-Las-Peñas et al., 2022; Frontera et al., 2022; Sahanic et al., 2022; Ziauddeen et al., 2022; Reese et al., 2023). A cluster with a lower number of overall symptoms and either predominant anosmia or no characteristic symptoms has been reported in four studies (Kenny et al., 2022a; Fernández-de-Las-Peñas et al., 2022; Frontera et al., 2022; Whitaker et al., 2022), and finally a neuropsychiatric cluster with symptoms such as brain fog, depression and insomnia has also been reported in four individual studies (Canas et al., 2022; Caspersen et al., 2022; Danesh et al., 2022; Reese et al., 2023). In electronic health record based studies, laboratory abnormalities tend to cluster together (Zhang et al., 2022a; Reese et al., 2023). Whether these emerging phenotypes are impacted by elements such as vaccination and infecting variant is less well studied, but early evidence suggests infecting variant may affect clinical phenotype, while vaccination seems to play a less important role (Kenny et al., 2022b; Canas et al., 2022). Similarly, patients may transition across phenotypes over the course of their long COVID illness (Davis et al., 2021). A summary of studies using unsupervised clustering of long COVID phenotypes is shown in **1**. While this phenotyping approach to studying long COVID is becoming increasingly common, there is also a large body of work looking at organ-specific symptoms and their possible underlying pathologic mechanisms. Table 1.

**TABLE 1:** Studies using unsupervised statistical methods to identify long COVID phenotypes.

Author	Study population	Type of analysis	Symptom clusters/Phenotypes
Canas et al. (2022)	N = 9,323, 6,454 (69%) F, 16 (0.002%) hospitalised	Multivariate time-series Clustering based on Principle Component Analysis method	Three clusters across all variants: 1) Cardiorespiratory 2) Central neurological 3) Multiorgan symptoms
Caspersen et al. (2022)	N = 774, 449 (58%) F	Factor analysis	Two factors: 1) Neurocognitive 2) Cardiopulmonary
Danesh et al. (2022)	N = 411, 295 (66.9%) F, 124 (30%) hospitalised acutely	K medoids algorithm	Two clusters: 1) Neuropsychiatric 2) Pulmonary
Fernández-de-Las-Peñas et al. (2022)	N = 1,969, 915 (46%) F, 1,969 (100%) hospitalised acutely	K means clustering	Three clusters 1) Few symptoms with little functional impairment 2) Many symptoms, predominant shortness of breath, more functional impairment 3) Many symptoms, intermediate functional impairment
Frontera et al. (2022)	N = 122, 40 (33%) F, 122 (100%) hospitalised acutely	Hierarchical clustering	Three clusters 1) Few symptoms 2) Many symptoms including headache and joint pain 3) Primarily shortness of breath
Kenny et al. (2022a)	N = 233, 173 (74%) F, 75 (32%) hospitalised acutely	Multiple correspondence and Hierarchical clustering	Three clusters: 1) Many symptoms, predominantly pain/musculoskeletal. 2) Cardiorespiratory 3) Less symptomatic
Reese et al. (2023)	N = 2256, 1403 (62%) F, 440 (19%) hospitalised acutely	Specificity-weighted fuzzy phenotype matching and k-means clustering	Six clusters: 1) Multisystem and laboratory, 2) Pulmonary 3) Neuropsychiatric 4) Cardiovascular 5) Pain/fatigue 6) Multisystem and pain
Sahanic et al. (2022)	N = 2050, 1363 (66%) F, 0 (0%) hospitalised acutely	Partitioning around medoids	Three clusters: 1) Few symptoms, predominantly smell and taste symptoms (“Hyposmia/anosmia positive”) 2) Mainly fatigue (“Hyposmia/anosmia intermediate”) 3) Many multiorgan symptoms (“Hyposmia/anosmia negative”)
Whitaker et al. (2022)	N = 28,713, 18,109 (63%) F, 408 (1%) hospitalised acutely	Partitioning around medoids	Two clusters 1) Few symptoms, fatigue most common 2) Respiratory predominant
Zhang et al. (2022a)	N = 34,605, 20,656 (60%) F, 14,112 (41%) hospitalised acutels	Topic modelling and hierarchical clustering	Four clusters: 1; Cardiac and renal 2; Respiratory, sleep and anxiety 3; Musculoskeletal and nervous system 4; Gastrointestinal symptoms and rash
Ziauddeen et al. (2022)	N = 2550, 1363 (66%) F, 0 (0%) hospitalised acutely	Hierarchical clustering	Two clusters: 1) Cardiopulmonary 2) Multisystem symptoms (pain, neurocognitive, cardiopulmonary and systemic symptoms)

Legend: N = number of participants, F = female.

### Organ specific sequelae of COVID-19

Common organ specific symptoms and sequelae of long COVID are summarised in Table 2. Severe COVID-19 is characterised by pneumonitis leading to acute respiratory distress syndrome (ARDS). As in other aetiologies of ARDS, direct pulmonary injury can lead to fibrosis or persistent inflammatory changes on lung imaging in the post-acute period, and abnormal pulmonary function in terms of restrictive patterns on spirometry or impaired diffusion capacity (Gao et al., 2021; Vargas Centanaro et al., 2022). In a subset of individuals, severity of pulmonary fibrosis post COVID-19 may necessitate lung transplant (Roach et al., 2022). In addition to fibrotic changes, COVID-19 may also result in damage to the pulmonary vasculature even in the absence of parenchymal abnormalities. These include pulmonary embolism-like perfusion defects, or disseminated patchy perfusion defects, that may reflect a widespread angiopathy (Remy-Jardin et al., 2021). Pulmonary embolism or angiopathy can lead to chronic thromboembolic pulmonary hypertension in some cases (Cueto-Robledo et al., 2022). However, abnormal respiratory radiological or functional findings are predominantly seen in survivors of severe acute COVID-19, while pulmonary function tests and routine imaging are often normal in those with a mild initial illness (Alba et al., 2021; Guler et al., 2021). Despite these normal findings, dyspnoea remains one of the most commonly reported long COVID symptoms across the spectrum of disease severity, whether measured by self-report, physician assessment, or increase in prescription of medications for dyspnoea such as bronchodilators in those with previous confirmed SARS-CoV-2 (Lund et al., 2021; Ziauddeen et al., 2022).

**TABLE 2:** Organ-specific phenotypes.

Organ system	Symptoms reported	References
Cardiorespiratory	Shortness of breath	Ziauddeen et al. (2022)
	Cough	Lund et al. (2021)
	Chest pain	Xie et al. (2022)
	Palpitations	Estiri et al. (2021)
Musculoskeletal	Joint pain	Stoffels et al. (2022)
	Myalgia	Huang et al. (2021a)
Neuropsychiatric	Brain fog	Taquet et al. (2021)
	Depression	Ceban et al. (2022)
	Insomnia	Huang et al. (2021b)
	Stroke	
Ear, nose and throat	Anosmia	Renaud et al. (2021)
		Wu et al. (2022)
Gastrointestinal	Change in bowel habits	Blackett et al. (2022)
	Nausea	Weng et al. (2021)
	Dyspepsia	Liao et al. (2022)
	Abdominal pain	
Dermatological	Rashes	McMahon et al. (2021)
	Pustules	Goyal et al. (2022)
	Hair loss	Chularojanamontri et al. (2022)
Multi-system	Fatigue	Townsend et al. (2020), Joli et al. (2022)
	Reduced exercise tolerance	Joli et al. (2022)

Legend: Examples of organ-specific and multi-system symptoms reported in long COVID.

Cardiac complications of SARS-CoV-2 infection have been noted since the beginning of the pandemic, with elevations of biomarkers of cardiac injury noted in an initial case series from Wuhan, China (Huang et al., 2020a). Since then a much broader array of long term cardiac complications have been observed. A case control study from the Veteran’s Affairs healthcare service in the United States of America found increased risks of ischaemic heart disease, pericarditis, myocarditis, arrhythmias and heart failure in the 30 days to 12 month period post COVID-19 compared to matched controls (Xie et al., 2022). Additionally, chest pain, palpitations and objective tachycardia are often reported (Estiri et al., 2021), and in one study, individuals recovering from COVID-19 were noted to have a relative tachycardia for a median of 79 days post symptom onset (Radin et al., 2021).

Musculoskeletal manifestations of long COVID include myalgia, joint pain and muscle weakness. Individuals admitted to the ICU may develop weakness comparable to critical illness myopathy from other causes, which has been associated with persistent impairments out to 5 years (Herridge et al., 2011; Soares et al., 2022). Female sex and corticosteroid use during acute illness have also been associated with muscle weakness up to 1 year after infection (Huang et al., 2021a). Muscle weakness has been described in those with a mild initial illness (Stoffels et al., 2022), but whether this reflects more than just deconditioning or muscle disuse has not been determined. The underlying cause and pathogenesis of muscle weakness is poorly understood, with viral myositis, ongoing inflammation, and deconditioning all being proposed.

New gastrointestinal symptoms including diarrhoea, constipation, vomiting, abdominal pain and heartburn have been reported in individuals recovering from COVID-19 (Blackett et al., 2022), and presence of these symptoms does not necessarily correlate with having had these symptoms acutely (Weng et al., 2021). Liver enzymes have been reported to be elevated in 25% of people hospitalised with COVID-19 at 1 month follow-up post-discharge, although these elevations are usually transient in the absence of concomitant liver pathology (Liao et al., 2022).

There is an excess risk of both incident diabetes and hyperglycemia in the 12 month period post COVID-19, with the degree of risk correlating to the severity of acute illness (Xie and Al-Aly, 2022). Elevated risks have been shown for both type 1 and type 2 diabetes, and a greater risk with COVID-19 than with other upper respiratory tract infections (Zhang et al., 2022b).

A registry of dermatologic findings in COVID-19 found skin rashes occurred for various durations, with morbiliform, urticarial and papulosquamous eruptions relatively short lived, while pernio was more frequently associated with a duration of >60 days (McMahon et al., 2021). Other manifestations including pustular dermatoses (Goyal et al., 2022) have been reported. Hair loss has been reported in 22% of hospitalised individuals at follow-up, regardless of acute oxygen requirement (Huang et al., 2021b). Telogen effluvium, a diffuse non-scarring hair loss disorder which typically lasts <6 months and is associated with stress, hormonal changes or medications (Hughes and Saleh, 2022) are the most common suspected aetiologies underlying hair loss, but alopecia areata and other forms of alopecia have also been reported (Chularojanamontri et al., 2022).

Neuropsychiatric complications in the months following SARS-CoV-2 infection have been frequently reported. Anosmia, dysosmia and taste disorders which are characteristic features of acute COVID-19, may persist for months after the initial infection (Zayet et al., 2021; Tan et al., 2022). Severity of initial infection is most strongly associated with increased risk of new neurological diagnoses, but higher incidences of neurological sequelae are reported across all categories of initial COVID-19 disease severity. Cerebrovascular events, including intracranial haemorrhage and ischaemic stroke, are increased in the post-infectious convalescence period, with the most marked risk in those with encephalopathy at the time of initial infection (Taquet et al., 2021). Cognitive dysfunction is a major long COVID symptom, and objective cognitive impairment has been found even in those without subjective impairment (Ceban et al., 2022). Interestingly, longitudinal follow-up has shown an increased incidence of both anxiety and depression at 12 months compared to 6 months from the initial diagnosis (Huang et al., 2021a). Additionally, there is increased prescribing of anxiolytic and anti-depressant agents, as well as analgesics, in the 6 months following a diagnosis of COVID-19 (Al-Aly et al., 2021). Although the association between initial disease severity and subsequent psychiatric diagnoses is less clear than with neurological disorders, the underlying pathogenesis remains unclear. Postulated mechanisms include direct viral cytotoxicity in the central nervous system as well as altered coagulation. It is likely that there are other contributors, especially to the increase in reported psychiatric symptoms, including consequences of living under pandemic restrictions, income security, and the psychological and psychiatric impact of ongoing, and in some cases debilitating physical ill-health.

## Proposed mechanisms of long COVID

**FIGURE 3:**
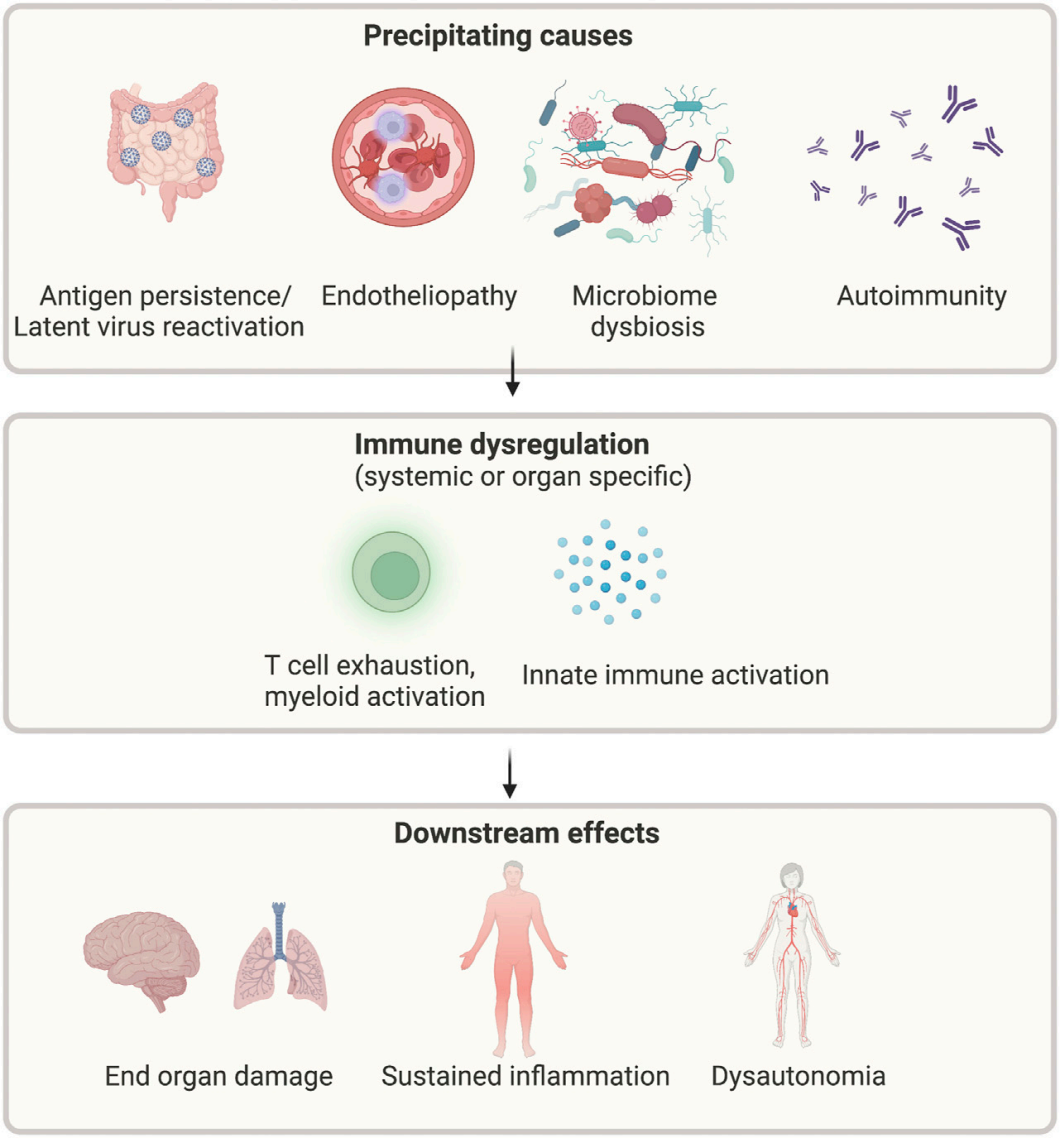
Proposed mechanisms of long COVID. Legend: Multiple possible precipitants, including persistent virus, reactivation of latent viruses, altered microbiome, endothelial dysfunction, and autoimmunity, may lead to ongoing immune activation and subsequent systemic symptoms. Created with BioRender.com.

There are multiple proposed pathogenic mechanisms that may contribute to the development of long COVID. These are summarised in Figure 3 and explained in greater detail below.

### Immune dysregulation

A small number of studies have examined changes in immune profiles or function following other acute viral infections. Expansion of activated CD8^+^ T cells have been reported in the setting of parvovirus B19 infection, as well as in the aftermath of severe influenza A H7N9 infection (Isa et al., 2005; Zhao et al., 2018). There is also increasing evidence of quantitative and qualitative changes in immune populations after SARS-CoV-2 infection. A prolonged period of T cell activation is seen in individuals, both hospitalised and non-hospitalised, after resolution of initial SARS-CoV-2 infection, with increased expression of the T cell exhaustion markers PD-L1 and TIGIT (Files et al., 2021). The persistent expansion of activated T cells appears most marked in older individuals (Townsend et al., 2022), which may in part be due to age-related immunosenescence and/or poorer T cell cross-reactivity between different coronaviruses in older individuals (Saletti et al., 2020). There is a similar reduction in naïve B cell numbers in convalescent samples from individuals with long-COVID-19 compared to recovered individuals or healthy controls (Phetsouphanh et al., 2022). Interestingly, blockade of PD-1 *ex vivo* can reverse the exhausted T cell state and restored T cell function, with increased responsiveness upon exposure to SARS-CoV-2 peptides (Loretelli et al., 2021). T cell exhaustion is demonstrated 1 year after infection (Taeschler et al., 2022). The mechanisms driving loss of T cell function post COVID-19, and how or if they contribute to the development of long COVID remains unclear. It may in part be due to the large number of viral-specific T cells that are produced during acute infection, especially in those with prolonged viral shedding (Moderbacher et al., 2020).

The immunological abnormalities seen following SARS-CoV-2 are not limited to lymphoid populations. There is evidence of increased innate cell activation, with myeloid cells displaying an activated phenotype (HLADR+CD38^+^), as well as production of type I and type III interferons. These abnormalities are most pronounced in patients with long COVID (Phetsouphanh et al., 2022). Persistent interferon abnormalities are perhaps not surprising, given the key role played by dysfunctional interferon responses in the development of severe acute COVID-19 (Bastard et al., 2021; Smith et al., 2022). Additional cytokines including IL-6, TNFα and IP-10 have also been shown to be elevated in long COVID (Peluso et al., 2021). Individuals with neurological features of long COVID, in particular brain fog, may have distinct circulating cytokines, including markers of blood brain barrier (BBB) disruption such as TGFβ and IL-8 when compared to those who have recovered fully from SARS-CoV-2 infection (Campbell et al., 2022). Specific inflammatory changes may be seen at the organ level. For example, individuals with persistent pulmonary parenchymal changes demonstrated both persistent upper airway type 1 interferon signalling and upregulated neutrophil associated signalling (George et al., 2022). A delayed but exaggerated interferon response in severe disease may promote neutrophil chemotaxis to lung tissue and the formation of neutrophil extracellular traps (NETs), which release proteases and cause extracellular membrane degradation, leading to fibrogenesis (George et al., 2022). A summary of the major immune changes described in long COVID are shown in Table 3. The aetiology of these immunological changes remains poorly understood, with antigen persistence, response to host tissue damage, or host factors in immune regulation all proposed as possible contributing factors.

**TABLE 3:** Immune cell and cytokine changes following SARS-CoV-2 infection.

Cell type	Changes described	Study population; median time since infection	References
T lymphocytes	Increase in activated T lymphocytes	111 patients, admitted and non-admitted; 101 days	Townsend et al. (2021c)
	Increase in markers of T cell exhaustion	59 patients; 6 months	Wiech et al. (2022)
B cells	Reduction in naïve B cell number	133 patients; 128 days	Phetsouphanh et al. (2022)
	Increased activated B cells	215 patients; 432 days	Klein et al. (2022b)
	Increased exhausted B cells	15 patients; 11 weeks	Jing et al. (2021)
Dendritic cells	Reduction in circulating DCs	71 patients; 7 months	Pérez-Gómez et al. (2021)
Macrophages	Increased pro-inflammatory phenotype	68 patients; 4 months	Bohnacker et al. (2022)
Pro-inflammatory cytokines	Elevated IL-6, TNFα and IP-10	121 patients; 124 days	Peluso et al. (2021)
	Elevated TGFβ and IL-8	32 patients; 146 days	Campbell et al. (2022)

Antibody responses to pandemic coronaviruses (SARS-CoV, SARS-CoV-2 and MERS) have been studied in detail. Most, but not all, individuals mount a measurable serologic response following infection with SARS-CoV-2 (Wajnberg et al., 2020), and while increased disease severity leads to a more robust serologic response (Huang et al., 2020b), even individuals with a mild initial illness can retain protective immunity against reinfection or development of severe disease from similar variants (Hall et al., 2021). Waning of serologic responses over time, as well as the emergence of new variants has meant that reinfection is now common, but development or recurrence of severe disease remains rare. There is ample evidence that quantitative neutralising antibody response correlates with protection against both re-infection and development of severe disease (Khoury et al., 2021) but a defined threshold of protection has yet to be determined. Similarly, vaccination has been associated with a lower risk of development of long COVID, but there are conflicting results regarding the magnitude of protection (Al-Aly et al., 2022; Antonelli et al., 2022), and it is unknown if there is a threshold of antibody that would provide protection against long COVID in the event of a breakthrough infection. Additionally, there is some evidence that vaccination in the convalescent setting may ameliorate symptoms, although reports are conflicting (Notarte et al., 2022). It is unknown why vaccination may prevent or improve long COVID symptoms. One hypothesis is that vaccination attenuates acute disease severity, and thus subsequent immune derangements and organ damage. A limitation to this hypothesis is that long COVID occurs frequently even in those with a mild initial illness (Maglietta et al., 2022). A second theory is that long COVID is due to a dysfunctional antibody response, which either fails to clear viral remnants or leads to the induction of self-reactive antibodies or proinflammatory antibodies. Vaccination prior to natural infection may prime the immune system and avoid this dysfunctional response. Findings in support of this proposed mechanism include the identification of an immunoglobulin signature in acute infection that predicts long COVID (Cervia et al., 2022), and that the antibodies induced by vaccination have different glycosylation patterns to those induced by infection (Chakraborty et al., 2022).

### Viral persistence and latent virus reactivation

Unlike DNA viruses, most RNA viruses cause acute infections with a relatively short period of host infectivity and subsequent recovery and host immunity. However, full or partial viral persistence in tissues has been demonstrated after the infectious period of a number of RNA viruses, including Polio, Chikungunya, Ross River and Measles viruses, and is implicated in prolonged or late complications of infection (Griffin, 2022). The clinical pattern of relapsing and remitting symptoms seen in long COVID has led to the hypothesis that viral persistence may also be contributing to the development of this condition (Brodin et al., 2022). A potential source of viral persistence is the gut, with SARS-CoV-2 nucleocapsid protein detected in intestinal biopsies 4 months after initial infection, even in mild cases (Gaebler et al., 2021). Similarly, viral RNA can be detected in the faeces of patients post-SARS-CoV-2 infection, even in the setting of negative nasopharyngeal PCR (Natarajan et al., 2022). There have also been reports of detectable viral spike protein in blood (Swank et al., 2022), circulating monocytes (Patterson et al., 2022), lung tissue (Ceulemans et al., 2021) and urine (Tejerina et al., 2022), weeks to months after initial SARS-CoV-2 infection, though correlation with persistent symptoms or development of long COVID phenotypes is variable. Furthermore in a cohort of predominantly individuals requiring hospitalisation, SARS-CoV-2 RNAemia in acute disease was found to be associated with a higher odds of subsequent subjective long COVID memory complaints, independent of acute disease severity. While viral persistence may serve a functional role in allowing for evolution of the humoral immune response, increasing the breadth and potency of circulating anti SARS-CoV-2 IgG (Gaebler et al., 2021), it may also lead to sustained innate immune activation or demonstrate molecular mimicry with host antigens leading to the induction of autoimmunity.

Reactivation of other latent viruses, such as EBV, has also been implicated in long COVID. EBV RNAemia in acute illness is associated with subsequent long COVID fatigue, cough and memory complaints. While EBV RNAemia is generally absent in long COVID (Peluso et al., 2022a), higher levels of anti EBV antibodies that are associated with lytic infection have been demonstrated in those with long COVID compared to those without (Gold et al., 2021; Klein et al., 2022a).

### Autoimmunity

Early development of neutralising antibody response after initial infection is associated with survival in acute COVID-19, but abnormal humoral responses have been implicated in the uncontrolled excessive immune activation seen in severe acute COVID-19. Multisystem Inflammatory Syndrome in Children (MIS-C), described in paediatric cases of SARS-CoV-2, is mediated by B cell autoantibody production secondary to T cell activation following exposure to superantigen, and has also been described in cases of severe acute COVID-19 in adults (Consiglio et al., 2020; Sancho-Shimizu et al., 2021). Autoantibodies against a variety of targets have been described in acute SARS-CoV-2 infection, including anti-nuclear antibodies, antibodies associated with myositis, systemic sclerosis and connective tissue diseases (Chang et al., 2021), and antibodies against various immunomodulatory proteins (Wang et al., 2021), including type I interferon autoantibodies (Bastard et al., 2020; Bastard et al., 2021). In addition, afucosylated anti spike IgG may promote excessive inflammation through recruitment of macrophages, NK cells and production of cytokines (Hoepel et al., 2021; Chakraborty et al., 2022).

Similarly, development of autoantibodies have been implicated in long COVID, though there are conflicting reports in the published literature. Autoantibodies directed towards a wide variety of targets have been reported in long COVID, including antibodies against angiotensin, ACE, and muscarinic receptors (Klein et al., 2022a; Sotzny et al., 2022; Thurner et al., 2022). Associations between autoantibody detection and specific symptoms in long COVID has not yet been demonstrated (Klein et al., 2022a). In addition, autoantibodies such as neutralising anti-interferon antibodies, generated in acute infection, may theoretically promote the development of viral persistence and therefore contribute to the development of long COVID. However, detection of these antibodies in the acute phase has been associated with persistent respiratory symptoms (Su et al., 2022), there is a low prevalence of anti-interferon antibodies in the post-acute period (Peluso et al., 2022b).

### Microbiome and microbial translocation

Viral persistence within the intestinal tract may also impact the gut microbiota and contribute to dysbiosis. The gut microbiome is significantly altered in individuals with acute SARS-CoV-2 when compared with uninfected matched controls. Changes include depletion of species with immunomodulatory roles within the gut such as *Faecalibacterium prausnitzii and Eubacterium rectale* (Yeoh et al., 2021). Furthermore, the degree of dysbiosis was associated with both initial COVID-19 disease severity and levels of circulating inflammatory cytokines. Interestingly, gut microbial diversity remains depleted 6 months after SARS-CoV-2 infection, with those with more severe initial infection having the most marked differences (Chen et al., 2022). In a cohort of hospitalised individuals, both microbiome composition at time of hospital admission and lack of recovery of microbiome composition were associated with the development of long COVID (Liu et al., 2022). Furthermore, the dysbiosis observed in acute SARS-CoV-2 is associated with increased intestinal permeability and subsequent translocation of bacterial and fungal products into the circulation, further contributing to systemic inflammation (Giron et al., 2021; Bernard-Raichon et al., 2022). Similarly, the dysbiosis observed in the convalescent setting may also contribute to increased intestinal permeability. Zonulin, a physiologic driver of intestinal permeability, and Beta-D-glucan, a marker of fungal translocation, have both been shown to be elevated in individuals with long COVID, and positively correlate with plasma concentrations of IL-6, TNFa, IP10, and negatively correlate with quality of life scores (Giron et al., 2022).

### Dysautonomia

Autonomic dysfunction has been described following multiple infections, but the underlying mechanisms remain elusive (Carod-Artal, 2018). Orthostatic intolerance occurs when an individual cannot maintain an upright position due to either sympathetic system over-activation or cerebral hypoperfusion. The typical features of dysautonomia occur as a result of catecholamine release and include tachycardia, hypotension, fatigue, palpitations, and syncope (Chen-Scarabelli and Scarabelli, 2004). There have been small studies examining the role of autonomic dysfunction in individuals with these long COVID symptoms. Tilt-table testing has demonstrated orthostatic intolerance in a small number of those affected (Jamal et al., 2022). However, post-COVID-19 fatigue has not been shown to be associated with autonomic dysfunction (Townsend et al., 2021a). Interestingly, findings of dysautonomia post-COVID-19 have been shown to correlated poorly with symptoms, but are associated with objective reduction in functional capacity on cardiopulmonary exercise testing (CPET) (Ladlow et al., 2022).

While correlation with all symptoms is poor, dysautonomia may be implicated in some individuals with a cardiorespiratory phenotype of post COVID-19. In one study that performed invasive CPET in individuals with persistent dyspnea more than 1 year post acute COVID-19 and normal pulmonary and cardiac imaging, exercise limitation was demonstrated to be due to impaired peripheral systemic oxygen extraction, rather than deconditioning or cardiopulmonary limitation (Singh et al., 2022). During exercise, production of local vasodilatory substances as well as sympathetic nervous system mediated vasoconstriction to non-exercising areas improves systemic oxygen extraction (Singh et al., 2021). In chronic fatigue syndrome, this process is impaired due to small fibre neuropathy affecting microvascular tone and causing microvascular shunting (Joseph et al., 2021). A similar mechanism of autonomic dysfunction may be at play in long COVID, given the similarities between these two conditions, but confirmatory studies are needed.

There are multiple possible aetiologies for post-COVID autonomic dysfunction. The ACE-2 receptor, which is widely expressed on endothelial cells lining blood vessels, is bound by SARS-CoV-2 (Hamming et al., 2004). Lower cortisol in individuals with long COVID compared to recovered controls suggests dysfunction of the hypothalamic-pituitary-adrenal axis (Klein et al., 2022a; Su et al., 2022). Persistent cardiac dysfunction and inflammation, which has been demonstrated in cardiac MRI studies (Puntmann et al., 2020), may also contribute.

### Endotheliopathy

Acute severe SARS-CoV-2 infection is associated with a distinct coagulopathy, characterised by markedly elevated D-dimers and increased thrombotic complications (Fogarty et al., 2020; Sakr et al., 2020). SARS-CoV-2 can also directly infect endothelial cells, which may further contribute to coagulopathy (Varga et al., 2020). Given the role played by coagulopathy in acute SARS-CoV-2 infection, there has been extensive investigation of coagulation parameters in the setting of long-COVID. Sustained disturbances in coagulation pathways have been identified in post-COVID patients, with up to a quarter of those assessed having an elevated D-dimer 4 months after initial infection (Townsend et al., 2021b) Additionally, those hospitalised with acute COVID-19 have been shown to have reduced fibrinolytic potential and demonstrate increased thrombin generation at a similar timepoint (von Meijenfeldt et al., 2021). Sustained endothelial cell activation has been shown up to 10 weeks after initial acute infection in both hospitalised and non-hospitalised individuals, with increased factor VIII and soluble thrombomodulin levels (Fogarty et al., 2022).

Von Willebrand Factor, a marker of endothelial cell activation, also remains elevated in these individuals. It has been hypothesised that these changes are due to persistent endothelial dysfunction, and may result in ongoing, low-grade thrombus formation in the microvasculature, contributing to the symptoms reported in those with long COVID.

Disruption of the endothelium at the BBB has been proposed as a possible pathological process driving neurocognitive symptoms of long COVID. The BBB is a collection of endothelial cells, astrocytes, microglial cells and neurons, and acts as a bridge between the blood and the central nervous system (Langen et al., 2019). The neurological consequences and cerebral microvascular injuries seen in acute SARS-CoV-2 infection are well-described (Mao et al., 2020; Lee et al., 2021). The SARS-CoV-2 spike protein has been shown to impair BBB function (Buzhdygan et al., 2020). The prevalence of persistent cognitive dysfunction following SARS-CoV-2 infection has been reported as being above 50%, in contrast to results from prior pandemic coronaviruses (Kwong et al., 2020; Taquet et al., 2021). The detection of SARS-CoV-2 within the cerebrospinal fluid (CSF) of individuals with COVID-19 further supports disruption of BBB integrity during acute infection (Espíndola et al., 2021). Neuro-invasion of SARS-CoV-2 may result in neuronal loss and subsequent neurological impairment, while secondary inflammation may further contribute. Indeed, there is increasing evidence of structural brain changes following SARS-CoV-2 infection, associated with cognitive decline (Douaud et al., 2022) and abnormalities reminiscent of post chemotherapy neuronal changes have been observed in a mouse model of mild to moderate COVID-19 (Fernández-Castañeda et al., 2022). It is important to acknowledge that these changes seen in endothelial cells and at the BBB have not been shown to be directly pathogenic. The above studies, while demonstrating correlation between viral markers, symptoms, and markers of endothelial dysfunction, do not demonstrate direct causation. This is an area that warrants further mechanistic investigation, as it is of therapeutic potential.

In addition to structural changes, alterations in cerebral bloodflow are seen in the aftermath of infection, with changes persisting up to 10 months after infection (Tian et al., 2022). This may in part be due to the systemic inflammatory response triggered by SARS-CoV-2, which may increase BBB permeability (Alam et al., 2020). In patients with neurological features of long COVID, in particular brain fog, there are increasing circulating markers of BBB disruption such as TGFβ and IL-8 when compared to individuals who have recovered fully from SARS-CoV-2 infection (Campbell et al., 2022).

## Therapeutics

The absence of clear pathogenic pathways to explain the myriad of presentations of long COVID has contributed to slow progress in the development of effective therapeutics. There remains no unifying treatment for long COVID. Both pharmacological and non-pharmacological therapies have been trialled for specific long COVID symptoms. The most common non-pharmacological interventions include physical and cognitive pacing for chronic fatigue and cognitive dysfunction. These have shown to have some improvement in functional outcomes (Bateman et al., 2021; O’Brien et al., 2022).

The potential persistence of viral reservoirs have led to suggestions that antiviral therapies such nirmatrelvir-ritonavir and remdesivir may be of benefit. Evidence from clinical trials including those being conducted by the STIMULATE-ICP group and others (Clinicaltrials, 2022) is awaited to clarify the role of these treatments in long COVID. The STIMULATE-ICP consortium in the United Kingdom are undertaking a large, randomised, platform clinical trial in long COVID, seeking to improve on the definition, the diagnosis and the management of long COVID. The planned interventions focus on community-based therapies as well as multi-organ MRI imaging, with a view to expanding in pharmacological therapies. Proposed therapeutics include anti-inflammatories such as colchicine, as well as anti-histamines.

The evidence supporting post-COVID endotheliopathy and coagulopathy, as well as the coagulopathy seen in acute infection, has stimulated trials on prolonged anti-thrombotic therapy. Use of the factor Xa inhibitor rivaroxaban in patients with high risk of thrombotic complications, based on predictive scoring tools and D-dimer levels, has been shown improve reduce the risk of venous thromboembolism 1 month after discharge (Ramacciotti et al., 2022). However, extended administration of these therapies beyond 1 month, their use in individuals who were not hospitalised with COVID-19, or impact on sequelae other than venous thromboembolism has not been extensively studied. Furthermore, the American Society of Haematology have issued a conditional recommendation against the use of anticoagulant prophylaxis post hospital discharge, although they acknowledge that there is very low certainty in the evidence and that high-quality, randomised controlled trials are needed (Cuker et al., 2022). The STIMULATE-ICP group hope to address the need for high-quality trial data by assessing response to rivaroxaban in this population (Forshaw et al., 2022).

The suggestion of ongoing immune dysfunction has also been the target of research into potential therapeutics for long COVID. Aberrant type-I interferon responses can be modulated by the histamine 2 receptor antagonist famotidine. This has shown earlier resolution in serum type-I interferon levels in patients with acute SARS-CoV-2 infection, and this was also associated with shorter time to symptom resolution (Brennan et al., 2022). However, to date there have been no RCTs examining the effect of anti-histamines in long COVID.

There has also been recent reports of positive impact of targeted therapeutics for management of persistent brain fog and cognitive dysfunction in long COVID. In a small case series, a combination of guanfacine, an α2 adrenoceptor agonist, and n-acetylcysteine, an anti-oxidant, was assessed in twelve individuals, eight of which reported symptomatic improvement (Fesharaki-Zadeh et al., 2023). Although preliminary, this pilot data can provide the basis for design of appropriately powered, placebo-controlled study to evaluate its efficacy.

Given the lack of evidence-based treatments, prevention of long COVID is a key to reducing the overall burden of this condition. Both vaccination against SARS-CoV-2 and certain modifiable lifestyle factors are associated with lower risk of long COVID-19 (Wang et al., 2023), and there is evidence that acute treatments including antivirals and metformin may be of benefit (Carfì et al., 2020; Xu et al., 2020).

### Future directions

The lack of understanding of the pathological basis for the range of manifestations of long COVID continues to hamper the development of effective therapies and remains a significant unmet clinical need. As outlined here, a large number of symptoms and underlying abnormalities have been reported, but consistent, robust evidence of causal association is lacking in most cases. Studies to date have been limited by a lack of consensus definition or classification of long COVID, a focus on individual symptoms rather than defined phenotypes, disproportionate inclusion of individuals with a history of initial severe, acute COVID-19 illness, under-representation of minority groups and paucity of longitudinal studies. Furthermore, local changes at an organ level may be found in the absence of a measurable systemic inflammatory response, and requires examination of specimens such as CSF and respiratory samples which have been less well studied. Improving care for this large cohort will require a coordinated, structured and comprehensive research programme encompassing epidemiologic, basic science, translational and clinical studies. Immune perturbations within affected organs, including the lungs, gastrointestinal tract and the central nervous system, the effects of disease-independent factors such as sociodemographic factors, socioeconomic status and lifetime stress on chronic inflammation, immune response to acute infection, and immune recovery in the aftermath of infection are all areas that require further investigation.

Given the scale and economic burden of long COVID (Kingdom OonsU, 2022), clinical trials should be performed in parallel with mechanistic studies. A significant coordinated, international, interdisciplinary approach will be required to improve our understanding and management of this condition. While challenging, this effort offers the opportunity to significantly advance our understanding of host, pathogen and environmental factors urgently needed to understand post infectious conditions beyond long COVID.
